# A Role for Topographic Cues in the Organization of Collagenous Matrix by Corneal Fibroblasts and Stem Cells

**DOI:** 10.1371/journal.pone.0086260

**Published:** 2014-01-21

**Authors:** Dimitrios Karamichos, Martha L. Funderburgh, Audrey E. K. Hutcheon, James D. Zieske, Yiqin Du, Jian Wu, James L. Funderburgh

**Affiliations:** 1 Schepens Eye Research Institute/Massachusetts Eye and Ear and Department of Ophthalmology, Harvard Medical School, Boston, Massachusetts, United States of America; 2 Department of Ophthalmology, University of Pittsburgh School of Medicine, Pittsburgh, Pennsylvania, United States of America; Wayne State University, United States of America

## Abstract

Human corneal fibroblasts (HCF) and corneal stromal stem cells (CSSC) each secrete and organize a thick stroma-like extracellular matrix in response to different substrata, but neither cell type organizes matrix on tissue-culture polystyrene. This study compared cell differentiation and extracellular matrix secreted by these two cell types when they were cultured on identical substrata, polycarbonate Transwell filters. After 4 weeks in culture, both cell types upregulated expression of genes marking differentiated keratocytes (KERA, CHST6, AQP1, B3GNT7). Absolute expression levels of these genes and secretion of keratan sulfate proteoglycans were significantly greater in CSSC than HCF. Both cultures produced extensive extracellular matrix of aligned collagen fibrils types I and V, exhibiting cornea-like lamellar structure. Unlike HCF, CSSC produced little matrix in the presence of serum. Construct thickness and collagen organization was enhanced by TGF-ß3. Scanning electron microscopic examination of the polycarbonate membrane revealed shallow parallel grooves with spacing of 200–300 nm, similar to the topography of aligned nanofiber substratum which we previously showed to induce matrix organization by CSSC. These results demonstrate that both corneal fibroblasts and stromal stem cells respond to a specific pattern of topographical cues by secreting highly organized extracellular matrix typical of corneal stroma. The data also suggest that the potential for matrix secretion and organization may not be directly related to the expression of molecular markers used to identify differentiated keratocytes.

## Introduction

The cornea forms the optical portal for light into the eye [1] and its integrity is critical for vision. The cornea derives its shape from the stroma, which contributes to as much as 90% of the total corneal structure, with the epithelial and endothelial layers comprising the remaining 10%. This highly ordered tissue has long been a subject of interest among scientists, as maintaining optical transparency of this dense connective tissue is essential for vision. Once the cornea is wounded or trauma occurs, the cornea may become permanently scarred, resulting in loss of visual acuity. Unfortunately, at that stage very few options are available. Currently the primary treatment used to correct stromal scarring is corneal transplantation.

Both full thickness and partial thickness corneal grafts are effective means of restoring transparency to scarred corneas, but this procedure relies on fresh donated cadaveric tissue. The supply of donor corneas has been adequate for the last decade in the United States, although at times there may be waiting lists for corneas at individual eye banks. Internationally, there is a great shortage of donor corneas. Recent changes in the U.S. Food and Drug Administration's regulations regarding corneal transplantation increase stringency regarding testing and safety. These changes will impact the supply and decrease the pool of eligible donors. The potential shortage of tissue has raised interest in generating corneal tissue ex-vivo. Identification of the cells and the culture conditions to produce this tissue, however, has presented challenges due to highly organized ultrastructure of the stromal tissue required to maintain the strength and transparency of the cornea.

The corneal stroma is comprised of over 200 lamellae, which are 1–2 µm thick each, made up of aligned, tightly packed collagen fibrils 36 nm in diameter [1]. This highly ordered arrangement of fibrils and the consistently small fibril diameter are considered essential to corneal transparency. Disturbance in the diameter and orientation of collagen can compromise vision, resulting in permanent loss of visual acuity. Following injury, quiescent keratocytes are activated into motile mitotic fibroblasts and then into a fibrotic phenotype, termed myofibroblasts. These cells secrete the fibrotic matrix that disrupts corneal transparency [2], [3]. Fibrotic stromal matrix is long-lasting and not readily converted to transparent stromal ECM in situ. There is, therefore, a strong interest in understanding the environmental cues that elicit normal and fibrotic tissue secretion by the keratocytes of the corneal stroma.

Numerous earlier studies reported that corneal stromal cells cultured in serum-containing media on impervious substrata rapidly become fibroblastic, losing the keratocyte phenotype and matrix synthesis [4]–[8]. In serum-free conditions, primary keratocytes maintain an in vivo phenotype [4], continuing to exhibit some properties of keratocytes through one or two population doublings [8]–[11]. However, after greater expansion, corneal fibroblasts lose their keratocyte morphology, gene expression, and the ability to organize a cornea-like extracellular matrix (ECM) in standard tissue culture.

Recently, Zeiske and his coworkers reported that HCFs, when cultured in a Transwell environment in the presence of ascorbate analogs, are capable of making and organizing a cornea-like ECM [12]–[14]. More recently, we observed that the growth factor TGF-ß3 augmented the amount of the ECM produced in vitro and stimulated its lamellar organization [15]. During a similar time frame Du and coworkers identified a population of cells in the adult corneal stroma with properties of adult stem cells [16]. These corneal stromal stem cells (CSSC) can be expanded in vitro, and in serum-free conditions they express genes and proteins typical of keratocytes [16], [17]. The CSSC cells do not generate abundant ECM in two-dimensional culture, but as free-floating pellets they produce abundant ECM containing stromal-like molecular components and regions of aligned collagen [17]. When the CSSC were cultured on an aligned nanofibrous substratum, they formed parallel lamellar ECM similar to that of adult stroma [18], [19]. On substratum of randomly oriented nanofibers or a planar film of the same material, on the other hand, CSSC secretion and organization of stroma-like matrix was significantly reduced [18]. It is clear from these experiments that topographic cues from the substratum exert a strong influence on the synthetic ability of CSSC. It is less obvious how the data regarding the Transwell culture system with HCF relate to the behavior of CSSC on nanofiber substrata. The purpose of the current study was to carry out a direct comparison of ECM produced by CSSCs and that produced by HCFs when both cell types were maintained under similar conditions. We report that CSSCs, like HCF, exhibit the ability to express and organize an ECM resembling that of the human corneal stroma when cultured on Transwell filter substrata. On examination, the filters revealed a surface of parallel, aligned grooves suggesting that they may provide topographic cues initiating stromal ECM synthesis similar to that of aligned nanofiber substrata.

## Materials and Methods

### Tissue

De-identified human corneas from organ donors were obtained from the National Disease Research Interchange (NDRI; Philadelphia, PA) or from Center for Organ Recovery and Education (CORE; Pittsburgh, PA). Use of de-identified tissue from non-living individuals is not human subject research under DHHS regulations 45CFR46 and exemption from the human subjects regulation was recognized in writing by the Institutional Review Boards (IRB) of both institutions. Ethical aspects of the research protocols were approved by the Committee for Oversight of Research Involving the Dead (CORID).

### Human Corneal Fibroblasts (HCF)

HCFs were isolated as previously described [12]. Briefly, using a razor blade, corneal epithelium and endothelium were scraped and removed. The stroma was cut into small pieces (∼2×2 mm) and put into 6-well plates (4 or 5 pieces/well). Explants were allowed to adhere and Eagle's Minimum Essential Medium (ATCC; Manassas, VA) containing 10% fetal bovine serum (FBS: Atlantic Biologicals; Lawrenceville, CA) was added. After 1–2 weeks of cultivation, the fibroblasts were passaged into a 100 mm cell culture plate. The cells were allowed to grow to confluence before being used in the culture system.

### Human Corneal Stromal Stem Cells (CSSC)

CSSC were isolated from donated human corneal tissue using a modification of our published procedures [16], [18]. Briefly, limbal stromal cells solubilized using collagenase digestion were initially cultured at clonal density (1×10^4^ cells/cm^2^) in stem cell growth medium [16] supplemented with 100 ng/ml cholera toxin (Sigma Chemicals, St. Louis, MO). Colonies of small polygonal cells were selected for further expansion, passaging each time at 1×10^4^ cells/cm^2^. Cultures were never allowed to reach confluence.

### Culture Conditions

Both cell types (HCF and CSSC) were plated on polycarbonate Transwell membrane inserts with 0.45 µm pores (Costar; Charlotte, NC) in 6-well plates at a density of 1×10^6^ cells per well. The density chosen was in agreement with our previously characterized model [12], [13], [15]. HCF and CSSC were cultured for 4 weeks in Eagle's Minimum Essential Medium supplemented with 0.5 mM 2-O-α-D-glucopyranosyl-L-ascorbic acid (Wako Chemicals USA, Inc.; Richmond, VA), 10% FBS and antibiotics, with or without 0.1 ng/ml TGF-ß3 (R&D Systems; Minneapolis; USA). CSSC were also cultured in Advanced MEM (Invitrogen; Carlsbad, CA) containing 0.5 mM ascorbate-2-phosphate (Wako) and antibiotics, as previously described [17] ± TGF-ß3 (0.1 ng/ml). At week 4, the constructs were collected and processed for immunofluorescence, RT-PCR, or SHG microscopy.

### ECM Characterization

#### Immunofluorescence

Immunofluorescence was performed as previously described [12], [13], [15]. In brief, samples were fixed in 4% paraformaldehyde. After fixation, the samples for indirect-immunofluorescence were incubated at 4°C overnight with primary antibody—type III collagen (1∶40: Southern Biotech; Birmingham, AL), type I collagen (1∶100; Abcam, Cambridge, MA) or type V collagen (1∶50; Novus Biologicals; Littleton, CO), diluted in 1% bovine serum albumin (BSA)+0.1%Triton-X. Samples were washed and then incubated overnight at 4°C with the corresponding secondary antibody—donkey anti-goat IgG (1∶200, type III collagen) or donkey anti-Rabbit IgG (1∶200, type I and type V collagen: Jackson ImmunoResearch; West Grove; PA)—diluted in 1% BSA+0.1%Triton-X in PBS. For direct immunofluorescence, samples were incubated overnight at 4°C in phalloidin-rhodamine (Invitrogen) diluted in 1% BSA+0.1% Triton-X in PBS. Phalloidin stains the f-actin filaments and allows for better visualization of the cells. TOPRO-3 iodide (1∶1000, Invitrogen), a marker of all cell nuclei, was used to counterstain all of the samples. Negative controls, where the primary antibody was omitted, were run with all experiments. Samples were washed, mounted with Vectashield Mounting Media (Vector Laboratories; Burlingame, CA), and observed and photographed using a confocal TCS-SP2 Leica microscope (Leica Microsystems; Bannockburn, IL).

#### ECM Thickness and Cell count

Total thickness of the construct was calculated using the z-series obtained from the TCS-SP2 confocal microscope during the visualization of the immunofluorescence. Cell number per unit area was quantified and extrapolated to total area of the culture [12], [15]. A minimum of 3 confocal z-series images were used for each condition, which were averaged, plotted and analyzed using Graph Pad-Prism v 5.0.

#### Second Harmonic Analysis

Second harmonic analysis was performed as previously described [18]. Paraformaldehyde-fixed samples were permeabilized for 10 minutes with 0.1% Triton-X 100 in PBS and nuclei were stained with SYTOX Green (Invitrogen), 5 µM for 10 minutes at room temperature. Images of organized unstained collagen were captured using second harmonic generated signals in a multiphoton scanning confocal microscope (Olympus FluoView MPE Multiphoton; Olympus America Inc.). The 780 nm output of a femtosecond sapphire laser was used and 400 nm backscatter light was collected using a 40× oil objective. Excitation wavelength for collagen was 830 nm, and Sytox was imaged using indirect fluorescence with excitation at 488 nm. Transwell surface features were imaged with a Jeol JSM-6330F Scanning Electron Microscope scanning electron microscopy (SEM) after sputter-coating a 5–10 nm coat of with gold/platinum.

#### Quantitative Reverse Transcription PCR (qPCR)

RNA was isolated using the RNeasy and transcribed to cDNA with random hexamers and qPCR was performed using SYBR® Green RT-PCR Reagents as previously described [17] and according to manufacturer's protocol for 20 µl reactions. The gene copy number in each sample was determined from a standard curve for each target gene using dilutions of purified cloned plasmid DNA containing the target sequences. A similar quantification was carried out for of 18S RNA which was used to estimate the total RNA in each cell sample. Calculation of mRNA copies per ng cellular RNA were thus calculated and averaged to obtain mean +/− standard deviation (SD). Six genes were selected which have previously been used to identify keratocyte phenotype. Keratocan (KERA) [5] and prostaglandin D2 synthase (PTGDS) [20], are keratan sulfate core proteins. Corneal N-acetylglucosamine-6-O-sulfotransferase (CHST6) [21] and beta1,3-N-acetylglucosaminyltansferase-7 (B3GNT7) [22], are enzymes involved in keratan sulfate synthesis. Corneal crystallin, aldehyde dehydrogenase 3A1 (ALDH) [23], and aquaporin 1 (AQP1) [24], a transport protein, are cell-associated proteins, both highly expressed in keratocytes. A list of primers used is given in Table S1.

#### Statistics

All experiments were repeated at least three times and data were analyzed for significant variations (p<0.05) using the Student's t-test and Dunnett's Multiple Comparison test.

## Results

### Differentiation of Corneal Cells to Keratocytes During Culture

**Figure 1:**
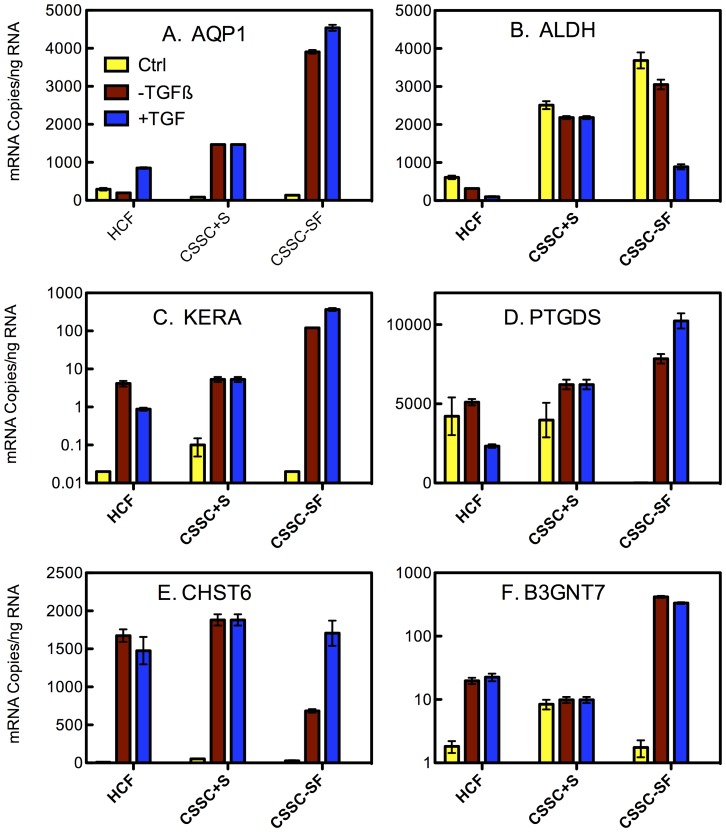
Expression of keratocyte genes by HCF and CSSC. The absolute mRNA abundance in cells before culture (yellow) and after 4 weeks of culture without TGF-ß3 (red) or with TGF-ß3 (blue) was determined for 6 genes associated with keratocyte phenotype (ALDH3A1, AQP1, B3GNT7, CHST6, KERA, PTGDS) as described in Methods. Error bars show standard deviation of triplicate assays. Some error bars are too small to be visualized on the plot.

Gene expression phenotype has been a useful tool in identifying the transition from stem cells to keratocytes [16]–[19], [25]–[28]. After 4 weeks in culture, expression of 6 genes marking keratocyte differentiation was compared between the two cell types using qPCR. As shown in Fig. 1, abundance of mRNA for genes representing keratocyte markers (except ALDH) increased significantly during the culture period. Genes involved in synthesis of the iconic corneal keratan sulfate (KERA, CHST6, and B3GNT7) were markedly upregulated during culture in both HCF and CSSC. In the case of KERA, the level increased more than 10,000-fold in CSSC (Fig. 1C) compared to uncultured cells as controls. PTGDS, a keratan sulfate proteoglycan protein, appears only to be upregulated in the serum-free differentiation conditions. In addition, AQP1, a transport protein abundant in keratocytes, was upregulated in both CSSC and HCF during the culture. Direct comparisons of the mRNA levels at the end of culture showed that CSSC in serum-free conditions expressed the highest levels of all the differentiation marker genes. CHST6 was an exception in that this mRNA was expressed at a similar level by all the cell types. Surprisingly, TGF-ß3 treatment had relatively little consistent effect on the overall expression level of these mRNAs.

**Figure 2:**
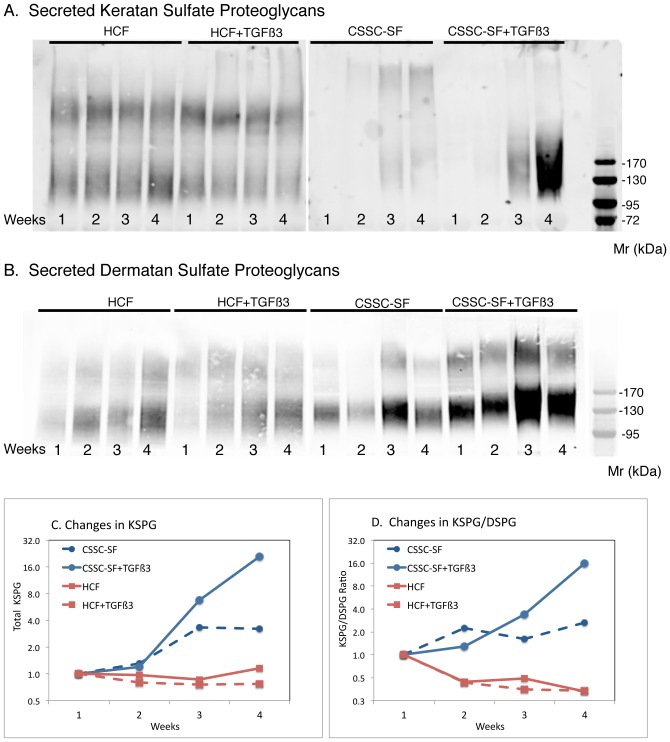
Secretion of corneal proteoglycans during culture. Proteoglycans isolated weekly from pooled culture media were detected by immunoblotting with (A) J19 [7], [16] monoclonal antibody for keratan sulfate or (B) BE123 [19] antibody against chondroitin/dermatan sulfate, as described in Methods. The total signal in each KSPG band (C) was quantified and plotted to show change relative to the amount at week 1. (D) KSPG/DSPG ratio was calculated from analysis of the samples on the same blot probed with the two different antibodies. TGF-ß3 cultures, solid lines; CSSC cultures, red lines; HCF cultures, blue lines.

Secretion of high molecular weight keratan sulfate proteoglycans (KSPG) is a unique molecular signature distinguishing keratocytes from other mesenchymal cells. In Fig. 2, a time course of KSPG secretion was examined by immunoblotting with antibodies to sulfated keratan sulfate. HCF culture medium contained material reacting with anti-keratan sulfate antibodies which did not change in abundance or size during the course of culture nor in the presence of TGFß3. KSPG in CSSC cultures, on the other hand, increased during the time in culture and was markedly stimulated in the presence of TGF-ß3 (Fig. 2A). Quantitation of these trends is shown in Fig. 2C. In the same samples, secretion of dermatan sulfate-containing proteoglycans (DSPG) was found to increase moderately in both cell types (Fig. 2B). When KSPG was normalized to DSPG secretion, the relative abundance of KSPG in HCF cultures was seen actually to decrease by 60% during culture, whereas KSPG secretion by CSSC increased more than 10-fold (Fig. 2D)

### ECM Accumulation

**Figure 3:**
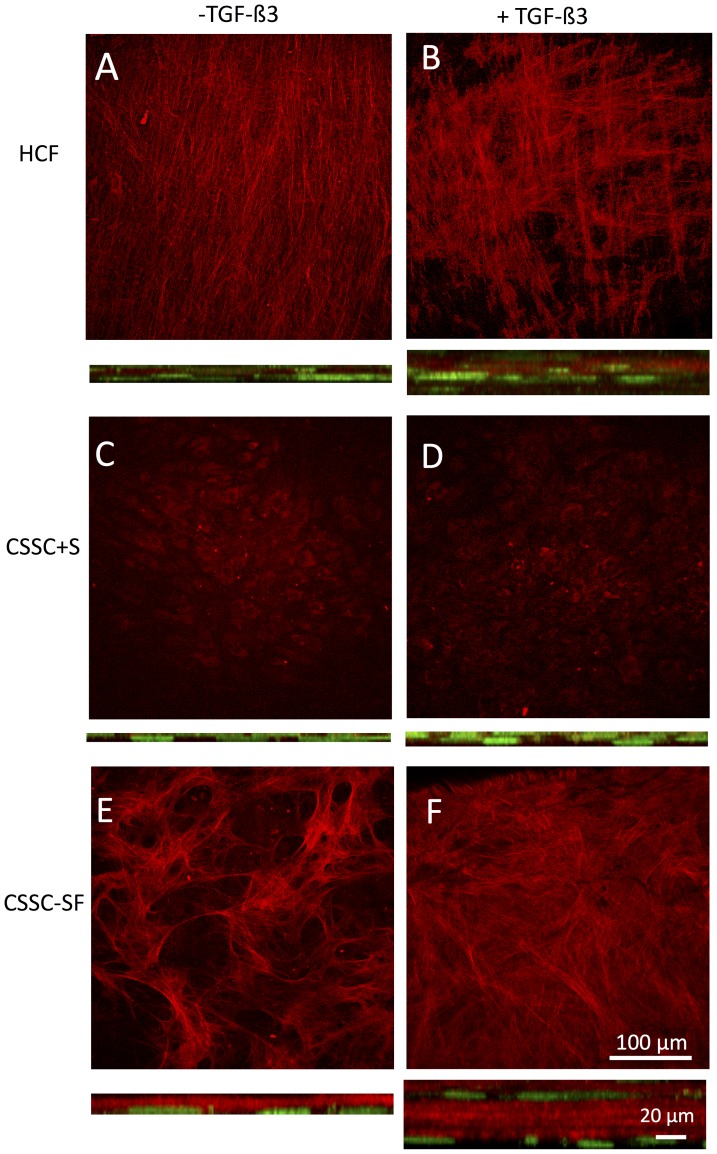
Second harmonic generation images of cell constructs. Images generated by fibrillar collagen were collected by excitation at 830–4×2 µm optical sections from a series of images through the entire construct. Below each image is an image representing a 100 µm×100 µm area of the full thickness image stack rotated on the X-axis. The height of these images provides representation of the thickness of the constructs. Cell distribution is visualized by Sytox Green staining of nuclei appearing green. (A, B) HCF (C, D) CSSC+Serum and (E, F) CSSC–serum free. Cultures were carried out without TGF-ß3 (A, C, E) or with TGF-ß3 (B, D, F).

Both HCF and CSSC produced obvious thick tissue-like constructs during culture. We compared the collagen organization in this material using SHG microscopy with a 2-photon confocal microscope. Fibrillar collagen produces SHG signals in response to irridiation with 800 nm light that can be imaged directly as a 400 nm fluorescence signal without need of immunostaining. Fig. 3 presents projections of z-stacks collected from reflectance SHG images each of the cultures. Fibrillar collagen secreted by HCF in the absence of TGF-ß3 showed uniform parallel alignment throughout the cultures (Fig. 3A). In the presence of TGF-ß3, the collagen bundles revealed orthogonal layers, indicating formation of lamellae (Fig. 3B). CSSC under the same conditions (Fig. 3C and D) generated very little signal, suggesting that these cells in the presence of serum organized little collagen. In serum-free conditions, however, the CSSC cultures in serum-free media (Fig. 3E) generated randomly oriented fibril bundles. In the presence of TGF-ß3 (Fig. 3F), the matrix appeared in orthogonal layers, similar to HCFs (Fig. 3B), indicating the formation of lamella. In the cross section projections, it is apparent that in the absence of TGF-ß3, cells were flattened and largely localized on the Transwell membrane at the bottom of each stack. In the presence of TGF-ß3, cells were clearly multilayered in all constructs. The most abundant fibrillar collagen was observed in the CSSC without serum (Fig. 3F) and in the HCF cultures (Fig. 3B).

**Figure 4:**
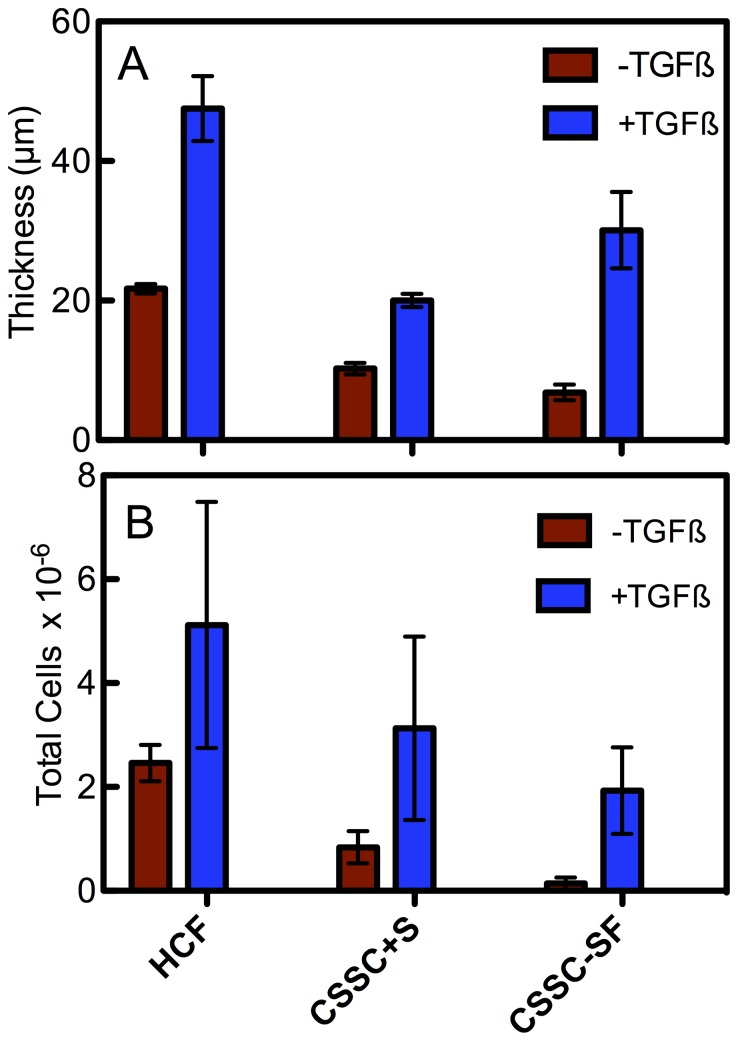
Construct thickness and cellularity. (A) Shows a graph of construct thickness at 4 weeks. TGF-ß3 treatment led to a significant increase in construct thickness for all conditions (*p<0.05). (**B**)_Graph of construct's total cells calculated from counts in optical sections of fixed cultures at 4 weeks incubation. Both cell types showed similar numbers ± TGF-ß3 when serum was present. CSSCs without serum had a significantly lower cell density (*p<0.05). Error bars = SD (n = 3).

Quantification of construct thickness in Fig. 4A shows that HCF generated constructs close to 45 µm in thickness, significantly greater than all of the other conditions. As seen in Fig. 3, all cultures produced significantly less tissue in the absence of TGFß3. The cell density of the constructs (Fig. 4B) with TGFß3 was similar in all three conditions. CSSC in serum without TGFß3, however, had significantly fewer cells.

### Collagen content of constructs

#### Type I Collagen

**Figure 5:**
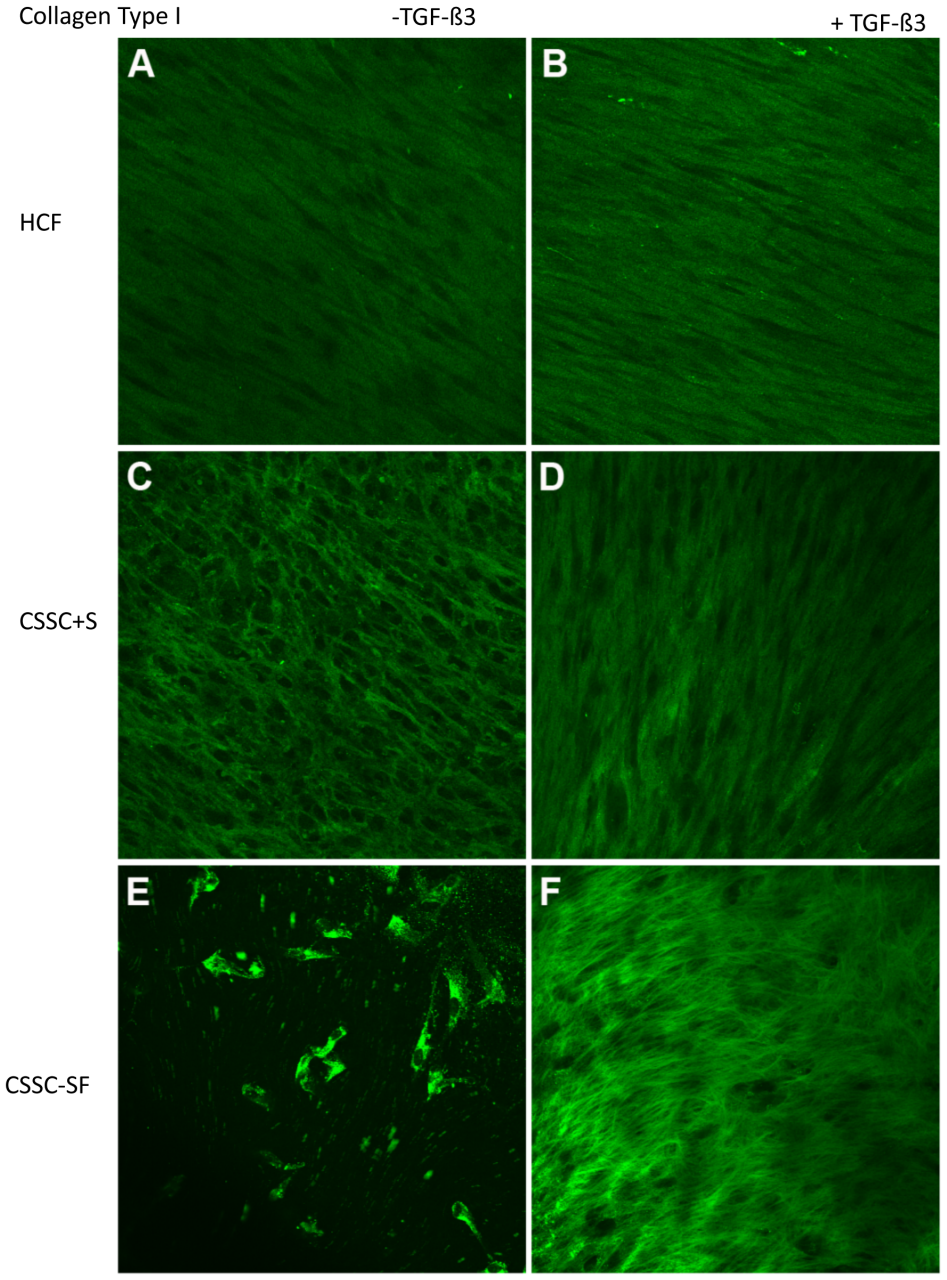
Collagen Type I in constructs. Confocal micrographs demonstrating immunolocalization of type I collagen at 4 weeks in (A, B) HCF, (C, D) CSSC in serum-containing media, and (E, F) CSSC in serum-free media. Constructs treated without TGF-ß3 are on the left (A, C, E) and with TGF-ß3 are on the right (B, D, F). Bar = 50 microns.

Type I collagen accounts for 85% of the fibrillar collagen in human corneal stroma. As seen in Fig. 5, type I collagen was present as aligned fibers in all of the cultures. Organization of the collagen was greater in cultures with TGF-ß3, marginally so with HCFs (Fig. 5A and B) and more pronounced with CSSCs (Figs. 5C and 5D). When CSSCs were cultured in serum–free media, type I collagen was present in the cells (Fig. 5E); however, with the addition of TGF-ß3, it was clear that an ECM of type I collagen was secreted with obvious parallel alignment (Fig. 5F).

#### Type V Collagen

**Figure 6:**
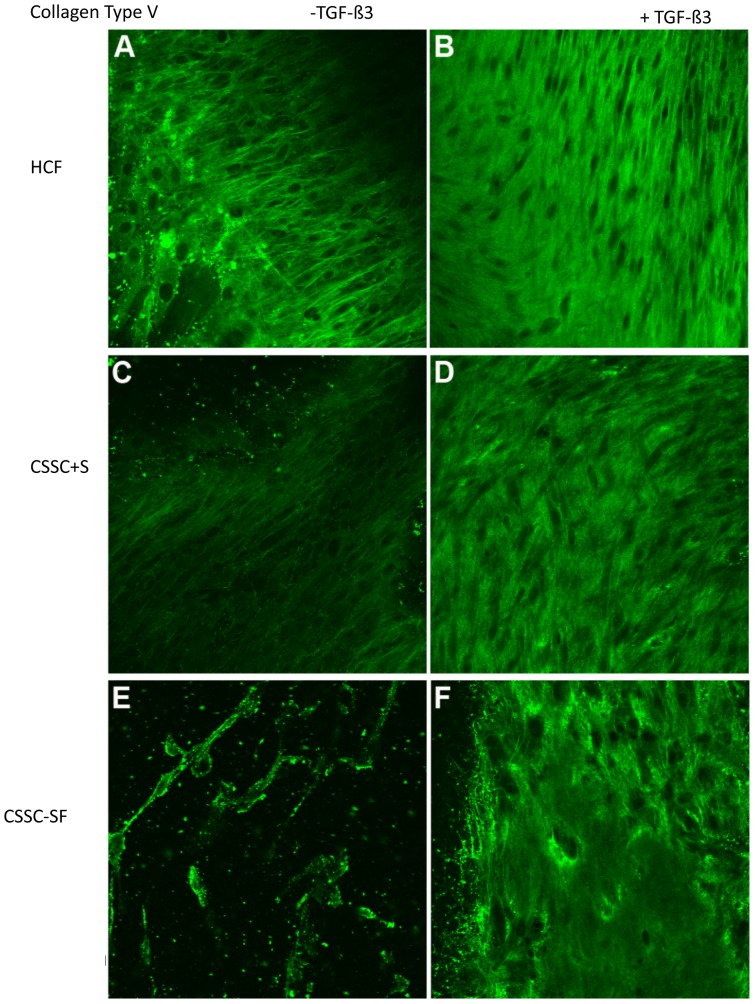
Collagen Type V. Confocal micrographs demonstrating immunolocalization of type V collagen at 4 weeks in (A, B) HCF, (C, D) CSSC in serum-containing media, and (E, F) CSSC in serum-free media. Constructs treated without TGF-ß3 are on the left (A, C, E) and with TGF-ß3 are on the right (B, D, F). Bar = 50 microns.

Type V collagen accounts for almost 10% of the corneal fibrillar collagen [29]. Similar to type I collagen, type V collagen was expressed under all conditions (Fig. 6), with cellular localization in CSSCs in serum–free media (Fig. 6E) and ECM localization with the addition of TGF-ß3 (Fig. 6F).

#### Type III Collagen

**Figure 7:**
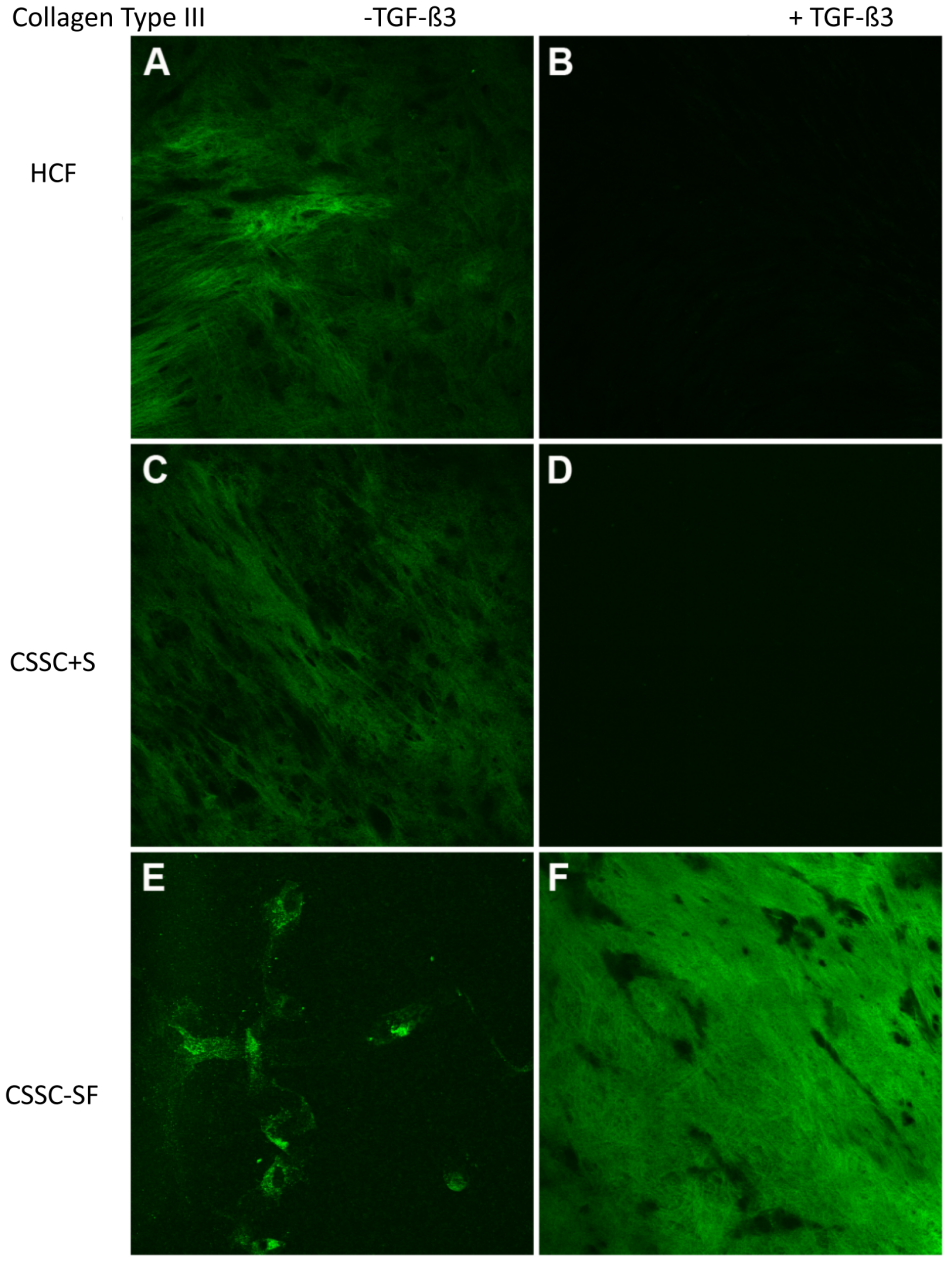
Collagen Type III. Confocal micrographs demonstrating immunolocalization of type III collagen at 4 weeks in (A, B) HCF, (C, D) CSSC in serum-containing media, and (E, F) CSSC in serum-free media. Constructs treated without TGF-ß3 are on the left (A, C, E) and with TGF-ß3 are on the right (B, D, F). Bar = 50 microns.

Type III collagen is fibrillar collagen not observed in normal corneal stroma, but present in corneal scars. It is considered a fibrotic marker. Both cell types had little to no expression of type III collagen (Fig. 7B and D) when cultured in the presence of TGF-ß3 and serum; however, when serum was absent, type III collagen was seen in the ECM produced by CSSC (Fig. 7E).

### Properties of the Transwell substratum

**Figure 8:**
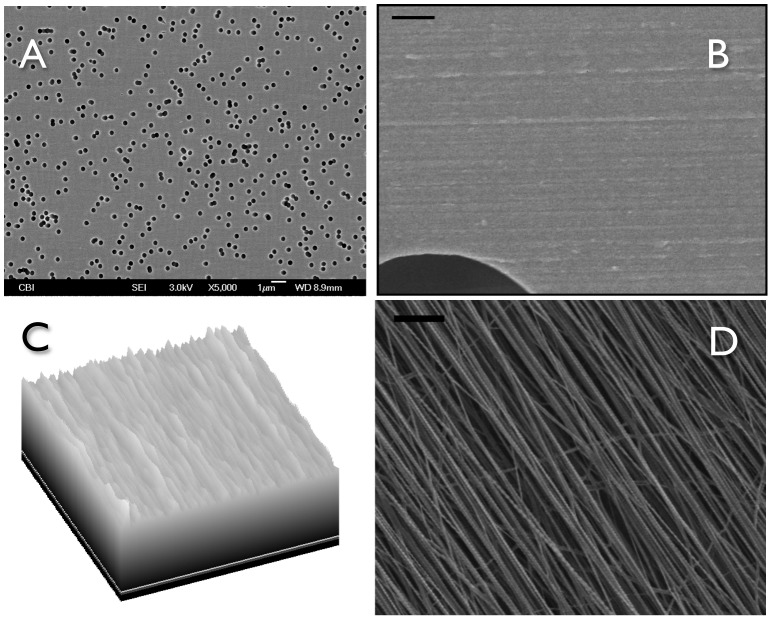
Ultrastructural Features of Polycarbonate Transwell Membranes. (A) Scanning electron micrograph (5000×) shows the 0.45 µm pores in the membranes. Scale Bar is 10 µm. (B) A 20,000× view shows parallel features on the membranes. Bar is 1 µm. (C) A portion of the image in (B) (0.5 µm×5 µm) was transformed using the “Surface Plot” algorithm of FIJI image analysis software to illustrate the regularity and spacing of the parallel features. Physical depth of the apparent grooves was not measured directly, but approximately 23 ridges are seen in the 5 µm section indicating an average spacing of 218 nm. (D) Electrospun PEUU nanofibers used as substratum for previous studies of CSSC average 165 nm in diameter [18], [19] and the spacing in this sample averaged 227 nm. Bar = 1 µm.

We previously showed that CSSC produce organized ECM in response to 3D features of the substratum [18]. In order to assess if Transwell filters provide a similar form of stimulus, we examined the surface of the Transwell filters using scanning electron microscopy. As shown in Fig. 8A, the Transwells presented as a planar surface randomly penetrated by 0.45 µm pores. In micrographs a higher magnification, however, Fig. 8B showed the presence of parallel linear surface features resembling shallow grooves on the membranes. Image enhancement analysis of the grooves, Fig. 8C, shows them to occur with about 250 nm spacing. This spacing is roughly similar to the architecture produced by the nanofiber substratum that induces ECM synthesis in the CSSC (Fig. 8D).

## Discussion

Stromal scarring and fibrosis limit vision for millions of individuals worldwide. Even though full or partial thickness keratoplasty is usually successful at reversing this blinding condition, donor tissue is not available to most individuals on the globe. Identifying alternate means to replace corneal tissue is one of the driving forces for corneal tissue engineering [30]. Some alternatives currently under investigation include non-biologic full thickness keratoprostheses and use of recombinant collagen for partial thickness grafts [31], [32]. The key for success in this endeavor is to find a strong transparent graft material that is not identified as a foreign substance and extruded by the eye.

Over the past decade, the idea that a bioengineered corneal graft, produced by, and populated with cultured corneal cells has gained credibility. Both CSSC and HCF have been shown to produce ECM in vitro closely resembling that of corneal stroma [12]–[15], [17]–[19]. These bioengineered tissue constructs currently lack the thickness and strength to translate to clinical trials, but they contain lamellae of corneal collagens with parallel orientation and small uniform diameters. These results represent a remarkable advance after decades of little progress and would appear to be an essential step required in the move toward a fully bioengineered stroma. An important element in this advance has been the use of cultures without rigid, impermeable glass or plastic substrata. In the case of the CSSC, some of us have shown that parallel aligned nanofiber substratum induces matrix organization, whereas random alignment or a cast-film of the same material does not [18]. This finding led to our hypothesis that linear, parallel, topographic cues from the substratum are required for inducing ECM secretion and organization by CSSC. HCF, on the other hand, secrete and organize stromal ECM when cultured on commercial polycarbonate Transwell filters [12]–[15]. Understanding the relationship between these apparently different culture environments was the motivation of this study.

There were clear differences between the two cell types when cultured on the Transwell membranes. To summarize: (1) CSSC did not perform as well in serum-containing medium as the HCF (Fig. 1, Fig. 3). (2) HCF generated a thicker ECM construct with a more aligned collagen than CSSC under the conditions chosen (Fig. 3, Fig. 4), and (3) CSSC expressed a higher level of keratocyte differentiation marker genes and more KSPG than HCF (Fig. 1, Fig. 2). These differences notwithstanding, the conclusion of the study is that both cells do produce a cornea-like ECM in the Transwell environment. This raises the question as to how the Transwell system might be providing the topographic cues that we found to be necessary for CSSC to generate corneal ECM [18]. To address that question we examined the surface of the Transwell filters using scanning electron microscopy. As shown in Fig. 8A, the Transwells presented as a planar surface randomly penetrated by 0.45 µm pores. In micrographs a higher magnification, however, Fig. 8B showed the presence of parallel linear surface features resembling shallow grooves on the membranes. Image enhancement analysis of the grooves, Fig. 8C, shows them to occur with about 250 nm spacing. This spacing is roughly similar to the architecture produced by the nanofiber substratum that induces ECM synthesis in the CSSC (Fig. 8D). We believe, therefore, that this undocumented feature of the membranes provides topographic cues to both cell types that are responsible for induction of organized corneal ECM in the Transwell system.

Nano-scale surface topology is well known to influence cell behavior. Particularly relevant are studies showing that substrata containing parallel grooves or ridges exert a marked influence in directing the differentiation of adult and pluripotent stem cells [33]–[38]. Aligned surface grooves have also been reported to influence the transformation of corneal fibroblasts to myofibroblasts [39] and to stimulate aligned collagen deposition by corneal fibroblasts but not dermal fibroblasts [40]. The studies with corneal fibroblasts used grooves of somewhat larger dimension (400–800 nm pitch) than the features we have identified [39]–[41]; therefore it will be important for future studies to define the dimensions and depth [42] of the topographical features that best stimulate the most effective matrix deposition. CSSC can already generate ECM constructs up to 80 µm thick. [19]. Optimization of topographic features of the substratum might help to achieve the goal of developing constructs useful for anterior lamellar keratoplasty or even deep anterior lamellar keratoplasty, this latter requiring tissue up to 500 µm in thickness.

The involvement of a member of the TGFß family is also interesting. TGFß1 and 2 are implicated in myofibroblast formation and generation of scar tissue [43]–[46], but TGFß-3 induces a response distinct from the other TGF isoforms, sometimes suppressing fibrosis and often inducing differentiation of stem or progenitor cells [15], [19], [30], [47]–[50]. The response to TGFß was recently shown to be sensitive to topographic features of the substratum [39]; therefore it is tempting to speculate that the 3D environment may contribute to the distinctly different effect of TGFß-3 compared to the other isoforms.

A second novel observation in this comparison of stem cells and fibroblasts is that both of these cell types generated somewhat equivalent tissue constructs in terms of lamellar structure and collagen alignment, in spite of the fact there were clear differences in the expression of markers for keratocyte differentiation. Three of the mRNA markers (KERA, B3GNT7, CHST6) relate to expression KSPG, a corneal-specific family of proteoglycans. HCF have long been known to lack KSPG synthesis [4], [6], [51], and immunoblot analysis (Fig. 2) confirmed that the concentration of KSPG decreased in HCF cultures during the 4 week experiment but increased in the CSSC cultures. Loss of keratan sulfate biosynthesis is responsible for macular corneal dystrophy in humans [52], [53], and in animal models loss of the KSPG protein lumican leads to defective to collagen fibrillogenesis and corneal haze [42], [54]. Additionally, corneal fibroblasts secrete ECM components, such as hyaluronan, fibronectin, biglycan, which are present in opaque corneal scars [7]. Consequently the ability of the fibroblasts to generate a collagenous ECM with similar properties to that in human stroma comes as somewhat of a surprise. Corneal fibroblasts derive primarily from stromal cells that have lost their distinctive keratocyte phenotype. Although early passages of corneal fibroblasts can undergo a partial reversion to the corneal phenotype [9], [11], after expansion in vitro these cells appear to have lost aspects of the potential to differentiate [8]. The reason that corneal fibroblasts regain differentiation potential is speculative at this point, but a recent report found that 3D environmental conditions can induce stem cell potential in fibroblasts [55]. We speculate, therefore, that some aspect of this culture system, distinct from tissue-culture polystyrene, allows the re-differentiation of fibroblasts to keratocytes. Because of the differences in proteoglycan synthesis, however, it remains to be determined if the tissue constructs produced by HCF will gain transparency in vivo. Our finding that corneal fibroblasts, like CSSC, respond to topographic cues on a nanometer scale, however, provides us new insight on the nature of keratocytes and offers additional evidence that corneal fibroblasts may be a useful cellular reagent in bioengineering of corneal tissue.

## Supporting Information

Table S1 **Primers used for real time qPCR.**
(DOCX)
