# Supplementary material for: A Role for Topographic Cues in the Organization of Collagenous Matrix by Corneal Fibroblasts and Stem Cells
Source: PLoS One. 2014 Jan 21;9(1):e86260. doi: 10.1371/journal.pone.0086260 (PMC3897697; doi:10.1371/journal.pone.0086260)
Supplement: Table S1 — Primers used for real time qPCR. (DOCX) [file pone.0086260.s001.docx]

| **Table 1.** Primers for real time qPCR | | |
| --- | --- | --- |
| Gene Symbol (Accession Number) | Direction | Sequence |
| ALDH3A1 (NM_001135168.1) | Forward | CATTGGCACCTGGAACTACC |
|  | Reverse | GGCTTGAGGACCACTGAGTT |
| AQP1 (NM_001185062.10) | Forward | CTGCACAGGCTTGCTGTATG |
|  | Reverse | TGTTCCTTGGGCTGCAACTA |
| B3GNT77 (NM_145236.2) | Forward | AAGAAAACCGTCTACCGGAGT |
|  | Reverse | TCAGCCAGAAATTCTAGCAGGT |
| CHST6 (NM_021615) | Forward | GAAATCCGTGCGCTCTACG |
|  | Reverse | GTCCAGATCCGTGGGTGAT |
| KERA (NM_007035.3) | Forward | ATCTGCAGCACCTTCACCTT |
|  | Reverse | CATTGGAATTGGTGGTTTGA |
| PTDGS (NM_000954.5) | Forward | CGGGGTCCCTCGGCTCCTAC |
|  | Reverse | CTGGGGGTCTGGGTTCGGCT |
| 18S rRNA (NR_003286.2) | Forward | CCCTGTAATTGGAATGAGTCCAC |
|  | Reverse | GCTGGAATTACCGCGGCT |
